# New paradigm for configurational entropy in glass-forming systems

**DOI:** 10.1038/s41598-022-05897-2

**Published:** 2022-02-23

**Authors:** Aleksandra Drozd-Rzoska, Sylwester J. Rzoska, Szymon Starzonek

**Affiliations:** Institute of High Pressure Physics of the Polish Academy of Sciences, Warsaw, Poland

**Keywords:** Physical chemistry, Statistical physics, thermodynamics and nonlinear dynamics

## Abstract

We show that on cooling towards glass transition configurational entropy exhibits more significant changes than predicted by classic relation. A universal formula according to Kauzmann temperature ${T}_{K}$ is given: $S={S}_{0}{t}^{n}$, where $t=\left(T-{T}_{K}\right)/T$. The exponent $n$ is hypothetically linked to dominated local symmetry. Such a behaviour is coupled to previtreous evolution of heat capacity $\Delta {C}_{P}^{config.}\left(T\right)=\left(nC/T\right){\left(1-{T}_{K}/T\right)}^{n-1}$ associated with finite temperature singularity. These lead to generalised VFT relation, for which the basic equation is retrieved. For many glass-formers, basic VFT equation may have only an effective meaning. A universal-like reliability of the Stickel operator analysis for detecting dynamic crossover phenomenon is also questioned. Notably, distortions-sensitive and derivative-based analysis focused on previtreous changes of configurational entropy and heat capacity for glycerol, ethanol and liquid crystal is applied.

## Introduction

Glass transition has remained a grand cognitive challenge of solid-state physics, chemical physics and material engineering for decades^1,2^. The hallmark feature is Super-Arrhenius (SA) previtreous behaviour of such dynamic properties as the primary relaxation time $\tau \left(T\right)$ or viscosity $\eta \left(T\right)$^2,3^:

1 $$\tau \left(T\right)={\tau }_{\infty }\mathit{exp}\left(\frac{{E}_{a}\left(T\right)}{RT}\right) \quad \eta \left(T\right)={\eta }_{\infty }\mathit{exp}\left(\frac{{E}_{a}\left(T\right)}{RT}\right)$$

where $T>{T}_{g}$, and ${E}_{a}\left(T\right)$ is the apparent activation energy. Basic Arrhenius behaviour is retrieved for ${E}_{a}\left(T\right)={E}_{a}=const$ in the given temperature domain. ${T}_{g}$ denotes glass temperature, which is empirically linked to $\tau \left({T}_{g}\right)=100$ s, and $\eta \left({T}_{g}\right)=1{0}^{13}$ P^4,5^.

**Figure 1:**
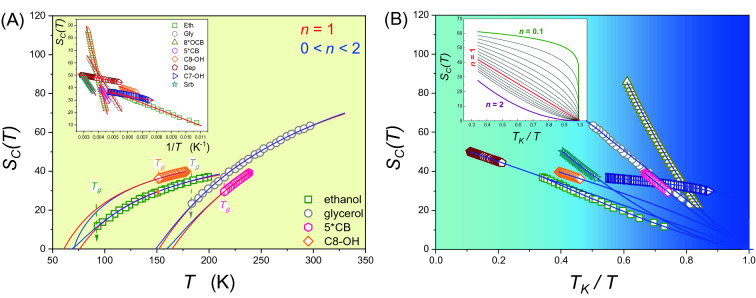
Configurational entropy for supercooled liquids. (**A**) Data portraying entropy behaviour for glycerol^57^, ethanol^58^, cyclooctanol^61^ and 5^*^CB liquid crystal^62^. Red and blue straight lines denote Eq. (5) and Eq. (10) respectively. Dashed arrows present glass transition temperatures *T*_*g*_. The insert shows configurational entropy as a function of reciprocal temperature ${S}_{c}\left(1/T\right)$ for all studied systems. (**B**) Configurational entropy normalised to the Kauzman temperature *T*_*K*_ for all samples basing on generalised Eq. (10). Impact of different *n* parameter on Eq. (10) is shown as an insert. Limit values *n* = 0.1 and *n* = 2 as well as classical case for *n* = 1 are marked by bold lines. Fitting parameters may be found in Table 1.

General SA portrayal of previtreous dynamics described by Eq. (1) has a rational meaning and cannot be used to parameterize experimental data, due to unknown form of activation energy ${E}_{a}\left(T\right)$^3^. Consequently, replacement relations must be applied. The dominant one is the Vogel-Fulcher-Tammann (VFT) dependence^2,6^:

2 $$\tau \left(T\right)={\tau }_{\infty }\mathit{exp}\left(\frac{{A}_{VFT}}{T-{T}_{0}}\right)={\tau }_{\infty }\mathit{exp}\left(\frac{{D}_{T}{T}_{0}}{T-{T}_{0}}\right)$$

where $T>{T}_{g}$, the amplitude ${A}_{VFT}={D}_{T}{T}_{0}=const$, ${D}_{T}$ is fragility strength coefficient, ${T}_{0}$ denotes extrapolated singular temperature ${T}_{0}<{T}_{g}$. The fragility ${\left[m=d{\mathit{log}}_{10}\tau \left(T\right)/d\left({T}_{g}/T\right)\right]}_{T={T}_{g}}$ is the key metric of the SA dynamics, indicating a deviation from the Arrhenius behaviour related to $m{}_{\min .} = \log_{10} \tau \left( {T_{g} } \right) - \log_{10} \tau_{\infty }^{{}} = 2 - \log_{10} \tau_{\infty }$. It is often estimated by the use of the fragility strength coefficient, namely: $m={D}_{T}{T}_{0}{T}_{g}/{\left({T}_{g}-{T}_{0}\right)}^{2}\mathit{ln}10$, and $m={m\left(1+\mathit{ln}10/{D}_{T}\right)}_{min}$^2,4,5^. The enormous popularity of the VFT relation, illustrated in Fig. 1, causes that it is often indicated as an empirical ‘universal’ scaling pattern for previtreous dynamics. Consequently, its derivations are often treated as a checkpoint for glass transition models^6–12^.

**Table 1:** Parameters calculated from the distortions-sensitive analysis.

Liquids	Abr	*T*_*g*_ (K)^a^	*n* = 1		*n* ≠ 1		
			*T*_*K*_ (K)	Δ*T* = *T*_*g*_* − T*_*K*_ (K)	*n*	*T*_*K*_ (K)	Δ*T* = *T*_*g*_* − T*_*K*_ (K)
Ethanol	Eth	93.6	77	16.6	1.28	68	25.6
Glycerol	Gly	183.5	152	31.5	1.04	149	34.1
Sorbitol	Srb	268.1	174	94.1	1.57	140	128.1
Cycloheptanol	C7-OH	130.1	48.5	81.6	0.16	116	14.1
Cyclooctanol	C8-OH	151.2	61	90.2	0.78	70	81.2
Diethyl phtalate	Dep	179.3	38	141.3	0.98	39	140.3
Isopentylcyanobiphenyl	5*CB	214.2	165	49.2	1.12	159	55.2
Isooctylcyanobiphenyl	8*OCB	221.2	203	18.2	1.51	185	36.2

The *T*_*K*_ was calculated directly from the Eq. (10), when the condition ${S}_{C}\left(T\right)\to 0\stackrel{}{\Rightarrow }{T|}_{{S}_{C}\left(T\right)=0}={T}_{K}$ is fulfilled.

^a^Glass temperature calculated for the relaxation time *τ* = 100 s.

The emergence of previtreous dynamics is associated with passing a melting temperature without crystallization and entering a metastable, supercooled domain^2,11,12^. In many ‘predominantly’ glass-forming systems, being of a particular interest of glass transition physics, supercooling is possible at any practical cooling rate, facilitating broadband dielectric spectroscopy (BDS) studies. In the previtreous domain, BDS requires frequency scans of electric impedance ranging from seconds to hours near ${T}_{g}$. BDS studies deliver high-resolution estimations of primary (α, structural) relaxation time from loss curve peak frequency $\tau =1/2\pi {f}_{peak}$. Previtreous changes of $\tau \left(T\right)$ are recognised as a basic characterization of previtreous SA dynamics^2–5,11,12^.

Configurational entropy (${S}_{C}$) is an essential thermodynamic characteristic of previtreous domain^2–5,8,9,11–22^. It describes a non-equilibrium entropy excess, taking entropy of equilibrium crystalline state as a reference. In 1948 Walter Kauzmann indicated that for some extrapolated temperature, hidden in a solid amorphous glass state one should expect ${S}_{C}\left(T\to {T}_{K}\right)\to 0$, usually 20–50 K below ${T}_{g}$^13^. The challenge associated with configurational entropy and the Kauzmann temperature ${T}_{K}$ explains the recent resume-report^20^: ‘*The configurational entropy is one of the most important thermodynamic quantities characterizing supercooled liquids approaching the glass transition. Despite decades of experimental, theoretical, and computational investigation, a widely accepted definition of the configurational entropy is missing, its quantitative characterization remains fraught with difficulties, misconceptions, and paradoxes, …practical measurements necessarily require approximations that make its physical interpretation delicate… the Kauzmann transition remains a valid and useful hypothesis to interpret glass formation. We also insisted that this is still a hypothesis but in no way a proven or necessary fact*…’.

Following above, for an ultimate cognitive insight into glass transition phenomenon, crucial may be reliable experimental evidence for ${S}_{C}\left(T\right)$ behaviour, matched to clearly non-biased estimation of ${T}_{K}$, and a non-ambiguous link to dynamics.

On the other hand, Berther et al.^20^ claimed, that: ‘*there is no, and that there cannot be any, unique definition of *${S}_{c}$*′*. However, based on author’s as well as other researchers’ best knowledge and experience, we decided to find a universality in configurational entropy behaviour. In the next part of the Report, we present a conventional definition of configurational entropy and its new critical-like description.

Experimentally, the configurational entropy may be estimated from an evolution of a heat capacity $\Delta {C}_{P}\left(T\right)$^2,12,15,16,20,21^:

3 $${S}_{C}\left(T\right)={\int }_{{T}_{K}}^{T}\frac{\Delta {C}_{P}\left(T\right)}{T}dT$$

where $\Delta {C}_{P}\left(T\right)={C}_{P}^{SL}-{C}_{P}^{glass}=\Delta {C}_{P}^{config.}$, with the heat capacity of glass instead of hardly detectable for ‘predominant’ glass formers, solid crystal entropy changes.

Assuming:

4 $$\Delta {C}_{P}\left(T\right)=\frac{\Delta {C}_{P}}{T}$$

with $\Delta {C}_{P}=const$, one obtains from Eq. (3) the ‘classic’, dependence for the configurational entropy^2,15,16^:

5 $${S}_{C}\left(T\right)={S}_{0}\left(1-\frac{{T}_{K}}{T}\right)={S}_{0}\left(\frac{T-{T}_{K}}{T}\right)={S}_{0}t$$

where $$t=\left(T-{T}_{K}\right)/T$$.

It is commonly used for describing changes of the configurational entropy in previtreous domain and an estimation of ${T}_{K}$^2,4,14–22^. One of the most inspiring models for glass transition was proposed by Adam and Gibbs (AG), five decades ago^8^. It links previtreous slowing-down to cooperatively rearranged regions (CRR), which influence configurational entropy, leading to following relation for previtreous changes of relaxation time^8^:

6 $$\tau \left(T\right)={\tau }_{\infty }\mathit{exp}\left(\frac{{A}_{AG}}{T{S}_{C}\left(T\right)}\right)$$

where ${A}_{AG}=const$ is the AG model amplitude.

**Figure 2:**
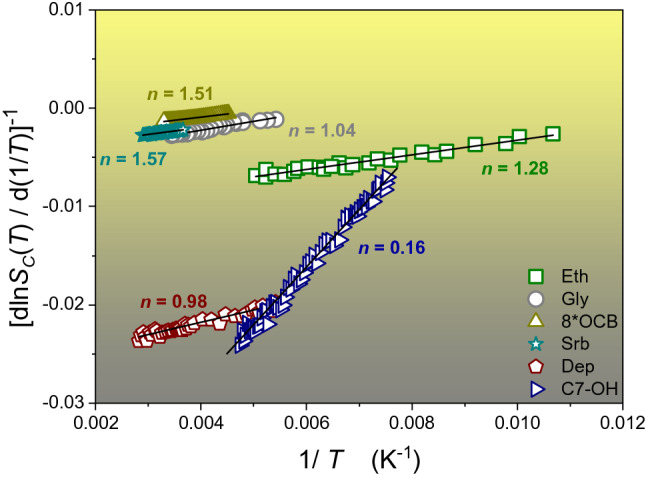
Distortions-sensitive analysis for the configurational entropy. Linearization ${S}_{C}\left(1/\mathrm{T}\right)=\mathrm{A}+Bx$, where $A=1/n{T}_{K},B=1/n$ Eq. (15). All calculated parameters *n* corresponds well with ones obtained using Eq. (10) (see Table 1).

Substitution of Eq. (5) into Eq. (6) yields the VFT relation, if ${T}_{0}\approx {T}_{K}$^2,8,12^. Numerous reports empirically support such a coincidence between a ‘dynamic’ and ‘thermodynamic’ singular temperatures for glass-forming systems^2,3,7–12,19–21^. Such an agreement also constitutes an essential reference for a set of theoretical models which link a finite temperature singularity in dynamics to a ‘hidden’ phase transition^2,3,7–12,19–21^. These empirical and theoretical correlations between ‘thermodynamic’ and ‘dynamic’ characterisations of previtreous domain, matched to enormous popularity of the VFT Eq. (2), significantly support Eq. (5) for describing configurational entropy and its usage as a tool for determining ${T}_{K}$. However, there are blots and non-coherences on the above landscape. Equation (5) poorly reproduce a variety of observed patterns for the heat capacity for $T\to {T}_{g}$ (see Fig. 2). As an empirical solution of this problem a relation $\Delta {C}_{P}^{conf.}\left(T\right)={\Delta {C}_{P}/T}^{\vartheta }$, with power exponent $0<\vartheta <2$ adjusted to a given glass former, was introduced^23^. However, it does not yield a coherent relation for configurational entropy and its model-basis is not clear. In 2003, Tanaka^24^ carried out validation tests of the VFT equation for 52 glass-forming systems and showed that $0.8<{T}_{0}/{T}_{K}<2.2$, i.e., the correlation ${T}_{0}\approx {T}_{K}$ appears only for a limited number of glass formers. There is also growing evidence questioning the omnipotence and a fundamental reliability of the VFT relation. It bases mainly on a comparison between experimental data and their scaling via VFT and other model relations. Subsequently, using visual or analytic-residual assessment of fitting quality, the VFT or other relations’ prevalence is tested. Nevertheless, observed discrepancies are subtle, occurring only in some temperature domains and they are close to an experimental error limit^2,11,12,25–29^. Consequently, such tests cannot yield decisive conclusions. Another type of validation of scaling relations is based on a superposition of $\tau \left(T\right)$ or $\eta \left(T\right)$ experimental data for a dozen glass-forming systems, using model-related parameters with individually selected (fitted) values for each tested system^2,11,12,30–33^. In the authors’ opinion, such a model-dependent scaling approach has tautological features and cannot lead to a breakthrough model-validation.

The recalled above record of puzzling results focused on confirming or rejecting the fundamental validity of the VFT relation had to be carried out for $T>{T}_{g}$, i.e., 20–50 K above singular temperatures (${T}_{K},{T}_{0}$). However, remote from singular temperatures, only subtle discrepancies between experimental data and model relations may be expected. An experimental error notably amplifies such a problem. Relatively strong discrepancies between experimental data and scaling relations can be expected only near hypothetical singular temperatures, i.e., in experimentally non-accessible domain.

To address mentioned inherent features of previtreous domain, an analysis concentrated exclusively on subtle distortions between a hypothetical scaling relation and experimental data may be decisive. In Refs.^34–36^. linearised derivative-based analysis focused on a portrayal via VFT^5,6,29,34,37,38^, MYEGA^27,35,36^, Avramov-Milchev^36,39^ and critical-like^40–42^ scaling relations were developed. For instance, the VFT parameterisation may validate a linear domain appearing in a plot based on the following transformation of $\tau \left(T\right)$ experimental data^34^:

7 $$\tau \left(T\right)\to {\left[\frac{d\mathit{ln}\tau \left(T\right)}{d\left(1/T\right)}\right]}^{-1/2}={\left({D}_{T}{T}_{0}\right)}^{-1/2}-{T}_{0}{\left({D}_{T}{T}_{0}\right)}^{-1/2}\times \frac{1}{T}=A-B\times \frac{1}{T}$$

Equation (7), in the form of the plot ${\varphi }_{T}=\mathit{ln}\tau \left(T\right)/d\left(1/T\right)$ vs. $1/T$, often named ‘Stickel operator’ analysis^43^, was used earlier for detecting a dynamic crossover temperature ${T}_{B}$, i.e., the crossover between ergodic and non-ergodic previtreous dynamical domains. The appearance of two lines in such a plot and their intersection related to ${T}_{B}$ are indicated as a ‘universal’ feature of previtreous domain^43–46^. Novikov and Sokolov strengthen this ‘universality’, suggesting a ‘magic’ time scale $\tau \left({T}_{B}\right)=1{0}^{-7\pm 1}$ s, estimated empirically by the ‘Stickel-operator’ analysis of 30 glass-formers, including low-molecular-weight liquids, polymers, ionic systems, covalent systems and plastic crystals^47^. However, some criticism regarding this finding appeared, due to glass formers with strongly different $\tau \left({T}_{B}\right)$ values^48^. Later, Roland showed a pressure–temperature invariance of $\tau \left({T}_{B},{P}_{B}\right)$^49^. It is worth nothing, that the linearised distortions-sensitive analysis showed that for glass-forming liquid crystals, plastic crystals and low-molecular-weight liquids with uniaxial molecules as well as a critical-like description are more reliable than the ‘classic’ VFT description^41,42^.

Hecksher et al.^50^ proposed to analyse previtreous dynamics using activation energy index ${I}_{DO}\left(T\right)=-d\mathit{ln}{E}_{a}\left(T\right)/d\mathit{ln}T=\left(d{E}_{a}/{E}_{a}\right)/\left(dT/T\right)$, i.e., to transform experimental data $\tau \left(T\right)\to {I}_{DO}\left(T\right)$. The required apparent activation energy was calculated using the general Super-Arrhenius Eq. (1), ${E}_{a}\left(T\right)=RT\mathit{ln}\left(\tau \left(T\right)/{\tau }_{\infty }\right)$, assuming a ‘universal’ value for pre-exponential factor ${\tau }_{\infty }=1{0}^{-14}$ s. In Ref.^50^ the analysis for 42 low-molecular-weight glass formers led to the conclusion: ‘…*there is no compelling evidence for the Vogel–Fulcher–Tammann (VFT) prediction that the relaxation time diverges at a finite temperature. We conclude that theories with a dynamic divergence of the VFT form lack a direct experimental basis*.’ However, results from Ref.^50^ might be biased by assuming a ‘universal’ value for the pre-factor, whereas experimental evidence suggests $1{0}^{-16}s<{\tau }_{\infty }<1{0}^{-10}s$^34,39^. In Ref.^51^, apparent activation energy was determined using a protocol avoiding this problem. It is based on a numerical solution of a differential equation directly resulted from the Super-Arrhenius Eq. (1) and applied for a given set of $\tau \left(T\right)$ experimental data^51^:

8 $$R\frac{d\mathit{ln}\tau \left(T\right)}{d\left(1/T\right)}=\frac{1}{T}\frac{d{E}_{a}\left(T\right)}{d\left(1/T\right)}+{E}_{a}\left(T\right)$$

The analysis of 26 glass-formers, including low-molecular-weight liquids, polymers, liquid crystals, colloids and even plastic crystals, revealed a common empirical pattern^51^:

9 $$\frac{1}{{I}_{DO}\left(T\right)}=a+bT$$

This result led to a general ‘empirical’ relation for the index^44,45^: $1/{I}_{DO}\left(T\right)=n{T}_{0}/\left(T-{T}_{0}\right)$, where ${T}_{0}$ is singular temperature determined from the condition $1/{I}_{DO}\left({T}_{0}\right)=0$ and the parameter $n=-1/a$. It was found that for tested systems $0.18<n<1.6$, and limits were related to domination of translational and orientational symmetries, respectively^51–53^. The previtreous dynamics described by the VFT relation is linked to $n=1$. Following mentioned results, a new relation for the configurational entropy was derived^51^:

10 $${S}_{C}={S}_{0}{\left(1-\frac{{T}_{K}}{T}\right)}^{n}={S}_{0}{t}^{n}$$

The ‘classic’ Eq. (5) is retrieved for $n=1$.

Problems of the VFT relation inspired the development of new scaling dependences for the previtreous dynamics. The leading position has gained Mauro-Yue-Ellison-Gupta-Allan (MYEGA) relation, which avoids the finite temperature singularity^27,35^:

11 $$\tau \left(T\right)={\tau }_{0}\mathit{exp}\left(\frac{C}{T}\mathit{exp}\left(\frac{K}{T}\right)\right)$$

Notably, it can be approximated by the VFT relation at ‘high-temperature’ domain^54^:

12 $${\mathit{ln}\left(\tau \left(T\right)/{\tau }_{0}\right)}_{=0}=\frac{C}{T}\mathit{exp}\left(\frac{K}{T}\right)=\frac{C}{T\mathit{exp}\left(-K/T\right)}\approx \frac{C}{T\left(1-K/T\right)}=\frac{C}{T-K}$$

where $K\approx {T}_{0}$, and $C\approx {D}_{T}{T}_{0}$, if comparing with VFT Eq. (2).

## Results and discussion

When discussing previtreous behaviour, one may consider substitution of Eq. (10) to the AG model relation Eq. (6). This yields a ‘generalised’ VFT relation:

13 $$\tau \left(T\right)={\tau }_{\infty }\mathit{exp}\left(\frac{{S}_{o}{A}_{AG}{T}^{n-1}}{{\left(T-{T}_{0}\right)}^{n}}\right)={\tau }_{\infty }\mathit{exp}\left(\frac{{A}_{VFT}{T}^{n-1}}{{\left(T-{T}_{0}\right)}^{n}}\right)={\tau }_{\infty }\mathit{exp}\left[\frac{{D}_{T}{T}_{0}/T}{{t}^{n}}\right]$$

where $t=\left(T-{T}_{0}\right)/T$. The ‘classic’ VFT formula (Eq. (2)) is retrieved for $n=1$.

Equation (13) has already been used for describing dynamics in glass-forming polyvinylidene difluoride (PVDF), PVDF + Barium-Strontium-Titanate (BST) microparticles composite^55^, and in its parallel form for describing relaxation time in relaxor ceramics^56^. Nevertheless, these tests cannot be considered as a crucial validation of Eq. (13) if recalling the above discussion. The milestone meaning could have derivative-based and distortions-sensitive tests focused directly on ${S}_{C}\left(T\right)$ experimental data. To fill such a cognitive gap a new solution is proposed in given report.

The analysis presented below explores state-of-the-art experimental results for the configurational entropy for 8 glass-forming liquids: glycerol^57^, ethanol^58^, sorbitol^59^, diethyl phthalate^60^, cycloheptanol^61^, cyclooctanol^61^ as well as liquid crystals^62,63^ (5*CB, 8*OCB). Basing on Eq. (10) one can propose the following distortions-sensitive transformation of experimental data:

14 $${S}_{C}\left(T\right)\to \mathit{ln}{S}_{C}\left(T\right)=\mathit{ln}{S}_{0}+n\mathit{ln}\left(1-{T}_{K}/T\right)\to d\mathit{ln}{S}_{C}\left(T\right)/d\left(1/T\right)=n{T}_{K}/\left(1-{T}_{K}/T\right)$$

Consequently:

15 $${\left[d\mathit{ln}{S}_{C}\left(T\right)/d\left(1/T\right)\right]}^{-1}=1/n{T}_{K}-1/nT=A+B\left(1/T\right)$$

Temperature dependence of the configurational entropy ${S}_{C}\left(T\right)$ of experimental data expressed by Eq. (15) should follow a linear behaviour, yielding optimal values for the reference Eq. (10): $n=1/B$ and ${T}_{K}=B/A$.

The characteristic feature of ‘generalised’ VFT Eq. (13) is power exponent *n*, influencing a distance from singular temperature distance ${T}_{0}$. Notably, a similar correction was advised in 1984 by Bengtzelius, Götze and Sjölander (BGS)^64^, basing on the mode-coupling theory, in 1988 by Bendler and Shlezinger (BS)^65^, using the mobile defects (‘random walk’) approach, as well as Hall and Wolyness^66^ for randomly packed spheres (HW):

16 $$\tau ={\tau }_{\infty }\mathit{exp}\left(\frac{F}{{\left(T-{T}_{K}\right)}^{\alpha }}\right)$$

where $\alpha \approx 1.76$ for BGS, $\alpha =3/2$ for BS, and $\alpha =2$ for HW models.

More recently, the random first-order transition (RFOT) model resulted in a similar dependence with an exponent $\alpha =\psi /\left(d-\theta \right)$^2^, where the exponent *d* is the spatial dimension, $\theta$ is for free energy surface cost on linear size of interface between two amorphous states and the exponent $\psi$ is a free energy barrier that must be overcome to rearrange a correlated volume. It is worth stressing that exponent $\alpha$ value, for mentioned models, is located within frames empirically indicated for the exponent *n*^51–53^.

Returning to the generalised Eq. (10) for configurational entropy, one can derive the relation for previtreous changes of the heat capacity, namely:

17 $$\Delta {C}_{P}^{config.}\left(T\right)=T\frac{d{S}_{C}}{dT}=\frac{n{S}_{0}{T}_{K}}{T}{\left(1-\frac{{T}_{K}}{T}\right)}^{n-1}$$

Heat capacity changes resulted from Eq. (17) are presented in Fig. 2, for the selected terminal, values of parameter *n*. Except the ‘classic’ case $n=1$, they show previtreous changes linked to a finite temperature singularity at ${T}_{K}$, which has been not expected for heat capacity so far. The insert in Fig. 2 recalls different heat capacity change patterns in a normalised scale for $T\to {T}_{g}$. To follow this issue, see also Refs.^67,68^.

**Figure 3:**
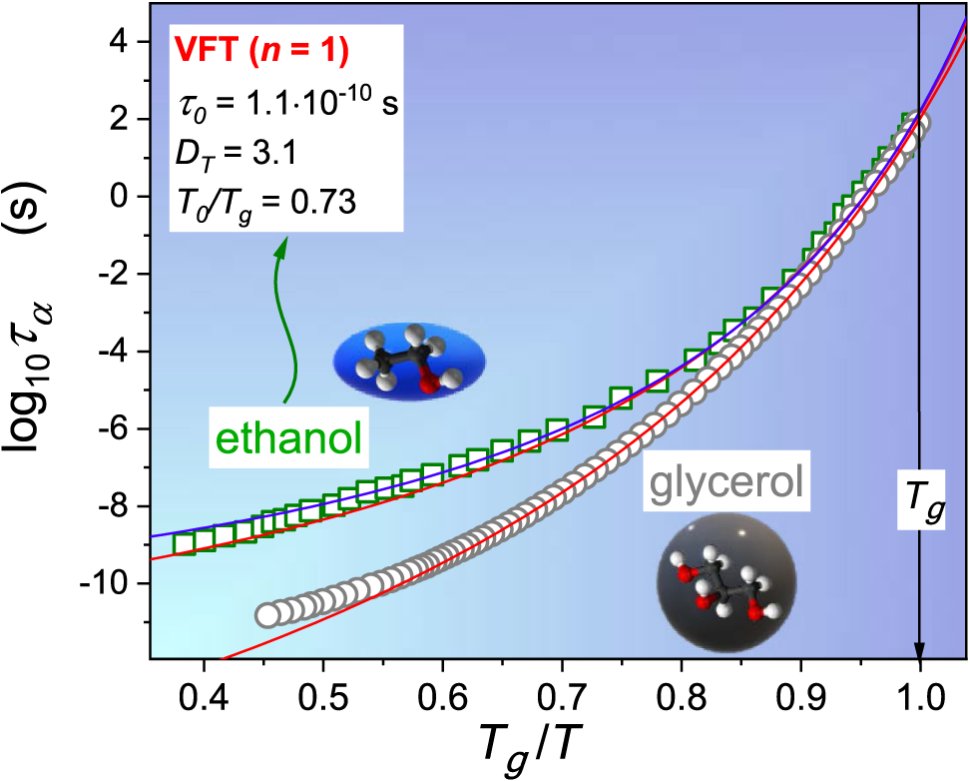
Evolutions of primary relaxation time for ethanol and glycerol presented in Angell plot. For glycerol ${T}_{g}=187.7K$ and ethanol ${T}_{g}=98.1K$. Experimental data in the central part of the plot are portrayed via the VFT Eq. (2) (in red) and the generalised VFT Eq. (13) (in blue), basing on the fitting domain $0.16<{T}_{g}/T<1$. Data obtained from Ref.^51–53^. Molecular structures taking from Wikimedia Commons.

One of glass transition experimental features is approaching the hypothetical Kauzmann temperature closer in heat capacity studies by increasing a cooling rate than in BDS tests for which the cooling rate factor is not important. Shifting below the standard $T{}_{g}$ value in DTA (differential thermal analysis) studies is often too strong ‘anomalous’ heat capacity changes. Such a behaviour via singularities appearing in Eq. (17). The description introduced by Eqs. (10) and (17) also correlates with recent indications for more pronounced changes of the configurational entropy than predicted by the classic Eq. (4) or indication for decoupling between VFT based estimations of the fragility (see comments below Eq. (2) and the real value of the fragility determined from the Angell plot (Fig. 3)^4,5,11,14–16^.

Notably, hypothetical validity of Eq. (17) opens a new possibility for distortions-sensitive tests directly exploring previtreous changes of the heat capacity:

18 $${\frac{d\mathit{ln}\left(T\Delta {C}_{P}^{config.}\right)}{d\left(1/T\right)}=\frac{{T}_{K}\left(n-1\right)}{1-{T}_{K}/T}\Rightarrow \left(\frac{d\mathit{ln}\left(T\Delta {C}_{P}^{config.}\right)}{d\left(1/T\right)}\right)}^{-1}={T}_{K}\left(n-1\right)-\frac{{T}_{K}^{2}\left(n-1\right)}{T}=A-\frac{B}{T}$$

The linear regression fit for a plot based on Eq. (18) may yield *A* and *B* coefficients, what gives consequently ${T}_{K}=B/A$, $n={A}^{2}/B+1$.

Figure 1 presents the configurational entropy evolution for supercooled glycerol, ethanol, sorbitol, cycloheptanol, cyclooctanol, diethyl phthalate, 5*CB and 8*OCB. Curves in the part A of Fig. 1 portraying experimental data, for selecting liquids, are related to the ‘classic’ Eq. (5) (in red) and the ‘generalised’ Eq. (10) (in blue). The Fig. 1A insert shows experimental data presentation based on a hardly explored scale ${S}_{C}$ vs. $1/T$, directly resulted from the Eq. (5). Figure 1B portrays configurational entropy normalised to the Kauzmann temperature *T*_*K*_ calculated from Eq. (10). The insert presents a behaviour of the Eq. (10) with different parameter *n*, i.e., $0.1<n<2$.

**Figure 4:**
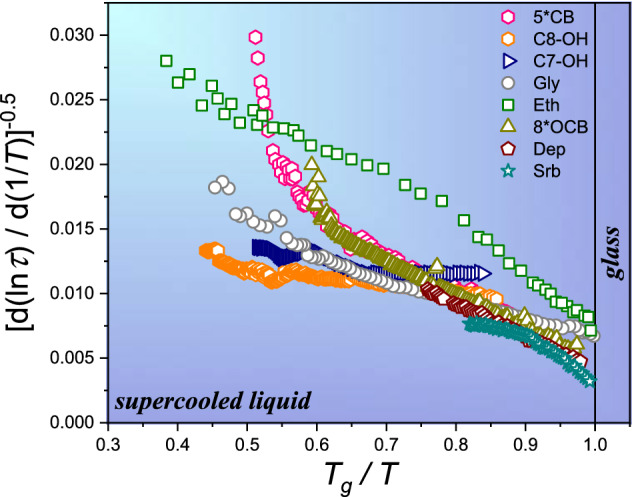
Linearised distortions-distortions sensitive analysis (‘Stickel plot’). Data are taken from the central parts of obtained results. Straight lines denote *n* = 1 and VFT description correctness. The temperature scale is normalised to *T*_*g*_.

Figure 4 presents results of the distortions-sensitive analysis of ${S}_{C}\left(T\right)$ experimental data based on Eq. (15). The linear behavior suggested by Eq. (15) appears, but with different slopes ($B\sim 1/n$). Obtained parameters for studied glass-forming liquids are collected in Table 1. These values are, within the limits of the experimental errors, the same as in Ref.^43^ e.g., $n=1.04$ for glycerol and $n=1.28$ for ethanol, which were obtained from the analysis of ‘dynamic’ experimental data $\tau \left(T\right)\to {I}_{DO}\left(T\right)$.

These results indicate that for glycerol and diethyl phthalate one can assume $n=1$, what leads to the VFT relation for relaxation time and the ‘classic’ expression for configurational entropy (Eq. (5)). On the other hand, for ethanol, sorbitol, 5*CB and 8*OCB the parameter $n>1$, what in Ref.^43^ was linked to glass former consisted of molecules with the uniaxial symmetry. One can expect that in such a case, the generalised VFT Eq. (13) may offer much more.

The main part of Fig. 3 presents previtreous behaviour of primary relaxation time in glycerol and ethanol using Angell plot^4,5^. Figure 3 shows the linearised distortions-distortions sensitive analysis of data from the central part of the plot, based on Eq. (7). Linear domains indicate the preference for describing $\tau \left(T\right)$ changes by the VFT relation (Eq. (2)). Such a behaviour is evidenced for glycerol but absent for ethanol.

**Figure 5:**
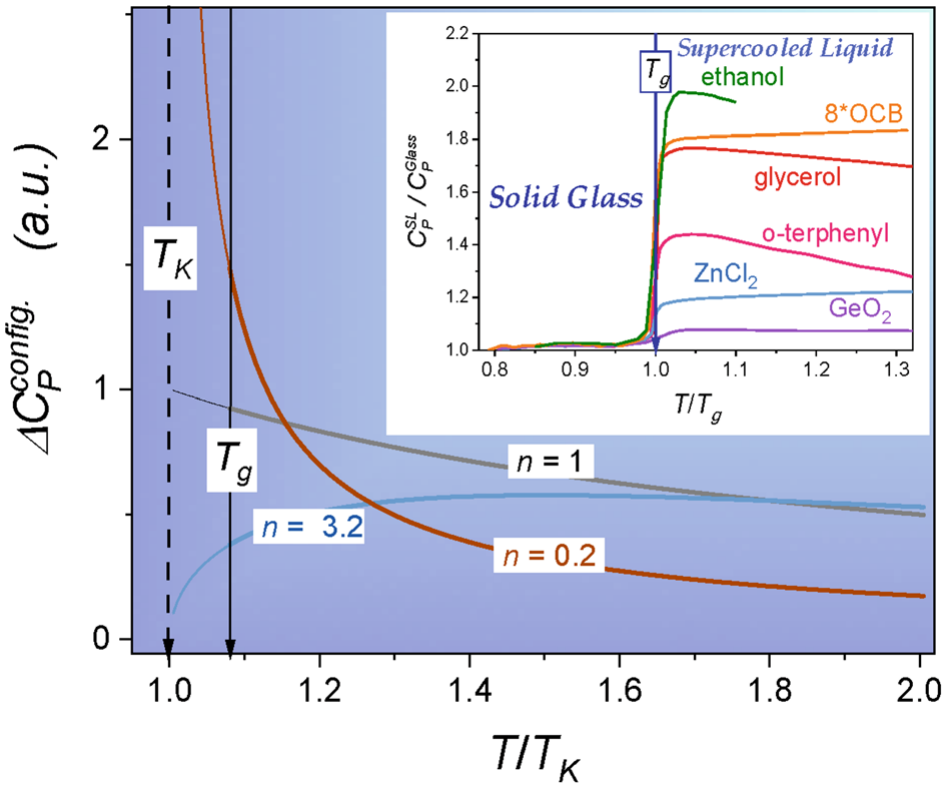
Previtreous temperature dependencies of configurational component of heat capacity in supercooled glass-forming liquids. The values resulted from Eq. (4), present different parameters *n*. Thin curves are for the extrapolation into the solid glass state. The inset shows examples of patterns of heat capacity changes for a selected cooling rate: prepared based on Refs.^67,68^.

Results related to Figs. 3 and 5 may be considered as the argument against the ‘universal’ validity of the ‘Stickel operator’ analysis used for testing dynamic crossover phenomenon^43–49^, due to inherently coupling to pre-assumption of an omnipotent validity of the basic VFT relation. The question also raised for general validity of discussions of fragility, i.e., the key metric for the SA dynamics of the previtreous domain^2,4,5^, within the context of recalled Eq. (2)^2,5,12,20,22,69–72^.

## Conclusions

Concluding, the report presents the evidence supporting the ‘generalised’ relation for the configurational entropy (Eq. (10)) and the protocol for linearised, distortions-sensitive analysis of related experimental data (Eq. (15)). All these may lead to deductions as follows:

- Configurational entropy ${S}_{C}(T)$ may be characterised by the critical-like behaviour, what gives corrected values of the Kauzmann temperature. Both are realised by the *n* parameter values similar to those calculated from dielectric data in the Dyre-Olsen energy index^51–53^.
- The ‘generalised’ relation for configurational entropy (Eq. (10)) also leads to the ‘generalised’ VFT Eq. (13). Its validity indicates the significance of testing the dynamic crossover phenomenon via the ‘Stickel operator’^43–49^ and problems of discussions focused on fragility within frames of the VFT relation^2,4,5,11,12,14,16,22,69–72^. Some discrepancies between the direct estimation of fragility and fragility strength by the use of VFT equation were raised recently^42,56^.
- Derivative-based analysis allows to neglect linear terms which may occur in the configurational expression if taking different values of heat capacity. This is a common problem in glass-forming liquids physics—what heat capacity should be chosen for the glass or the crystal state. However, because of the above, the configurational entropy may be calculated using the chosen solid phase.

The glass transition is most often indicated as the dominantly dynamic phenomenon, which heuristically supports impressive previtreous primary relaxation time or viscosity changes. This is supported by dependence of glass temperature and heat capacity behaviour from a cooling. This report proposed that the long-range, previtreous behaviour also occurs for such a basic thermodynamic property as configurational entropy and heat capacity. This may suggest not only dynamic but also thermodynamic character of glass transition.

## Methods^57^

DSC measurements were performed using a standard procedure for all studied liquids. When heat flow returns to the value zero, a new thermal equilibrium has been reached and next step is started. The procedure is suitable for heating and cooling. Assuming that the specific heat (*C*_*p*_) is constant in the small temperature range *ΔT*, it follows that ${C}_{p}=\left[\left({m}_{1}^{^{\prime}}+{m}_{2}^{^{\prime}}-{m}_{2}\right){\int }_{{T}_{1}}^{{T}_{2}}{C}_{p}^{Al}dT+\left({S}_{1}-{S}_{1}^{^{\prime}}\right){K}_{c}\right]/\left({m}_{1}\Delta T\right)$, where *S*_*1*_, is the area of the peak observed for the mass m, of liquid contained in an aluminium crucible of mass *m*_*2*_, and *S*_*1*_*′* is the area of the peak for mass *m*_*1*_*′* of aluminium contained in another crucible of mass *m*_*2*_*′*.

## Data Availability

All data are available after personal request.
